# Analog Front-End ASIC for Compact Silicon Photomultiplier Sensor Interfaces in Mixed-Signal Systems

**DOI:** 10.3390/s26020410

**Published:** 2026-01-08

**Authors:** Davide Badoni, Roberto Ammendola, Valerio Bocci, Giacomo Chiodi, Francesco Iacoangeli, Stefano Pasta, Gianmaria Rebustini, Luigi Recchia

**Affiliations:** 1 Istituto Nazionale di Fisica Nucleare Sezione di Roma Tor Vergata, Viale della Ricerca Scientifica 1, 00133 Rome, Italy; roberto.ammendola@roma2.infn.it (R.A.); stefano.pasta@roma2.infn.it (S.P.); gianmaria.rebustini@roma2.infn.it (G.R.); 2 Istituto Nazionale di Fisica Nucleare Sezione di Roma, Piazzale Aldo Moro 2, 00185 Rome, Italy; valerio.bocci@roma1.infn.it (V.B.); giacomo.chiodi@roma1.infn.it (G.C.); francesco.iacoangeli@roma1.infn.it (F.I.); luigi.recchia@roma1.infn.it (L.R.)

**Keywords:** SiPM front-end, analog ASIC, current-mode design, peak-and-hold, sensor interface, scintillator-based detector

## Abstract

We present a mixed-signal front-end ASIC designed for compact Silicon Photomultiplier (SiPM) sensor interfaces, implemented in the AMS 0.35 µm CMOS technology. The chip integrates two independent analog channels, each composed of five custom second-generation current conveyors (CCII^+^), a fast zero-crossing discriminator, and a peak-and-hold stage based on a tailored operational amplifier. The CCII^+^ and discriminator blocks were designed in-house, based on literature designs and adapted to the technology to ensure low input impedance and fast current-mode signal propagation. This architecture enables precise detection of small signals with reduced pile-up, important for time-resolved photon detection. Bias and threshold control are provided by programmable current mirrors and SPI-configurable DACs, including a 10-bit current-mode DAC based on a current-splitting structure with approximately 200 nA resolution. A custom SiPM behavioral model was developed in the Cadence environment to support design and simulation, reproducing realistic pulse shapes and recovery dynamics for timing applications. Circuit-level simulations confirm correct analog functionality and stable operation across the intended dynamic range, with a per-channel consumption of about 5.9 mA at 3.3 V (19.5 mW), reflecting a tradeoff between speed and robustness. The system is compatible with external timing architectures, while internal CCII^+^ stages ensure low-impedance current reception, fast discrimination, and accurate current-to-voltage conversion for peak detection.

## 1. Introduction

Silicon Photomultipliers (SiPMs) are solid-state photon sensors widely adopted in particle and photon detection systems thanks to their compactness, high gain, low operating voltage, and insensitivity to magnetic fields. When coupled to scintillators, SiPM-based detectors enable the simultaneous measurement of interaction time and deposited energy, which represents a core requirement in applications ranging from nuclear and particle physics to space-borne and compact instrumentation. Achieving accurate reconstruction of fast scintillation signals requires front-end electronics capable of handling sub-nanosecond current pulses, wide dynamic range, and high event rates while maintaining low noise and stable operation. Recent developments in integrated front-end ASICs for SiPM readout demonstrate a broad spectrum of architectural solutions addressing different trade-offs in fast amplitude processing, noise performance, and system integration, confirming the continuing relevance and diversity of this research field [1].

Meeting these requirements motivates careful architectural choices in the analog front end. In particular, the adoption of low input impedance at the sensor interface is a deliberate design choice to enable fast current-mode signal reception, mitigate pile-up effects at high counting rates, and preserve linearity over a wide dynamic range. Current-domain processing allows efficient handling of the fast SiPM avalanche current while minimizing voltage excursions at the sensor node. In addition, scintillator-based detectors require not only precise timing discrimination but also reliable peak amplitude capture to extract energy information. These considerations motivate the integration of fast discrimination, pile-up reduction, and peak-and-hold functionality within a compact and programmable analog front end, rather than relying on discrete components and heterogeneous signal paths. Integrated ASIC solutions combining fast discrimination with charge-based energy readout for SiPM-based detectors have been recently proposed in large-scale experimental contexts, highlighting different system-level trade-offs and application-driven priorities [2].

This work is developed within the LITE-SLPD (Lightweight Integrated Technology for Space Luminescence and Particle Detection)research program promoted by INFN, aimed at the progressive miniaturization and integration of SiPM-based detector systems for compact and exploratory applications. Within this context, the ArduSiPM platform has evolved through multiple generations, starting from early board-level implementations (GEN1) based on discrete analog front-end electronics and microcontroller platforms [3], followed by significant improvements in compactness, power consumption, and system integration in subsequent generations (GEN2 and GEN3) [4,5]. Despite these advances, previous realizations still relied on discrete analog circuitry, motivating the transition toward a fully integrated solution. Alternative mixed-signal ASIC architectures for SiPM sensor readout have also been reported in the literature, mainly addressing system-level timing architectures rather than detailed analog front-end implementation [6]. The present work represents a pilot step toward the forthcoming GEN4 architecture, whose primary objective is the complete integration of the analog front-end chain into a dedicated ASIC, replacing the discrete analog electronics used in earlier generations. The proposed dual-channel architecture is conceived to support future time-of-flight (TOF) developments in combination with segmented Time-to-Digital Converter (TDC) solutions, while the present prototype is primarily focused on the integration and validation of the complete analog front-end chain. The ASIC operates in conjunction with an external microcontroller/SoC for configuration and data acquisition and is designed to achieve a per-channel analog current consumption of approximately 5.9 mA at 3.3 V (19.5 mW), reflecting a balanced trade-off between speed, robustness, and suitability for compact detector systems. The prototype ASIC is implemented in a mature AMS 0.35 µm CMOS technology with a 3.3 V supply, deliberately selected for its robustness, cost-effectiveness, and fast prototyping turnaround. The complete dual-channel ASIC has been submitted for fabrication through an AMS 0.35 µm MPW run, with post-silicon characterization planned upon chip availability.

## 2. System Architecture Overview

**Figure 1:**
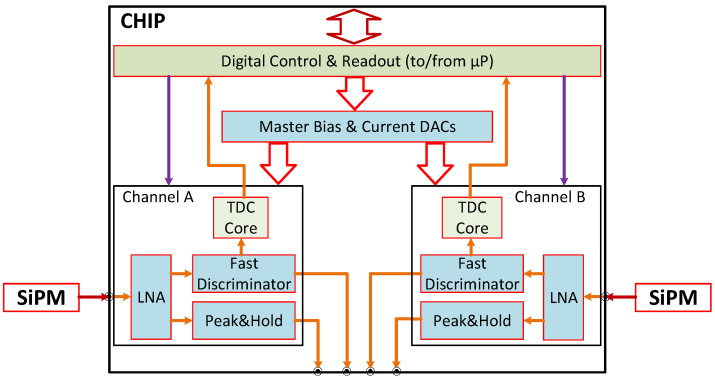
Block diagram of the ASIC architecture, showing the two analog channels, the embedded segmented TDC core, the common Digital Control & Readout block (to/from µP), and the Master Bias & Current DACs block distributing programmable references to the channels. Color coding is used to distinguish analog/mixed-signal channel circuitry from digital control, readout, and timing blocks.

The proposed ASIC integrates the full set of analog front-end functions required for SiPM signal processing following a current-mode readout approach commonly adopted in fast photon and particle detection systems, while introducing a compact and fully integrated implementation tailored to lightweight detectors. This all-in-one integration explicitly targets the reduction of external analog components and interconnections, a key requirement for compact and robust implementations in constrained environments such as space-borne and nanosatellite platforms. The architecture is organized in two independent channels, each including a low-noise current-mode input stage (CCII^+^), a fast zero-crossing discriminator, and a peak-and-hold (P&H) circuit (see Figure 1). The choice of a dual-channel architecture is also driven by the constraints of space-borne and nanosatellite applications, where compact all-in-one solutions are required to minimize mass, volume, and external components. This approach is fully aligned with the design philosophy of the LITE-SLPD experiment, which aims at highly integrated and lightweight detector systems.

Each channel also embeds a TDC core, implemented as a segmented delay-line structure triggered by the fast discriminator and referenced to a system clock derived from the external µP. In this first prototype, the TDC core is included mainly to validate the segmented delay-line concept and its integration within a compact mixed-signal ASIC, while the primary focus of the chip remains the integration and validation of the analog front-end functions. Timing words generated by the TDC core are collected and serialized through the common digital block.

Both channels share the Digital Control & Readout and the Master Bias & Current DACs blocks, which manage SPI-based register loading, global bias configuration, and the collection of TDC outputs. These blocks provide a global reference current used to bias the CCII^+^, set global source/sink injection levels, and control the local 10-bit DACs driving each discriminator threshold. While bias currents are distributed globally, the discriminator thresholds are independently programmable for each channel, ensuring flexibility in multi-channel operation and fine adjustment of detection sensitivity.

By combining fast triggering with peak amplitude capture, the ASIC provides both temporal and energy-resolved information from the SiPM. This dual capability directly reflects the core requirements of scintillator-based detectors, where precise interaction timing must be correlated with the deposited energy, while maintaining compatibility with photon-counting applications.

## 3. Analog Front-End Channel Design

### 3.1. Design Choices

The analog front-end follows a current-domain design philosophy, where input signals are received, conditioned, and discriminated entirely in current mode [7,8,9]. From the output of the first LNA stage, the signal is split into two paths: one feeding the fast discriminator, while the other feeds a current-to-voltage conversion stage, enabling subsequent amplitude extraction through the Peak-and-Hold circuit. This dual-path approach minimizes input impedance and supports high-speed operation, which are essential for SiPM readout [10,11]. The input stage exploits the low-impedance property of the X terminal of the CCII^+^, allowing direct current-mode coupling to the SiPM and minimizing voltage build-up at the sensor interface.

Fast timing is supported by a discriminator chain implemented in current mode, providing efficient pile-up reduction and single-photon sensitivity, while amplitude information is extracted by a peak-and-hold (P&H) circuit. Two operational amplifier configurations are employed in the P&H path: an Improved OPAMP, tailored for high-speed peak capture, and a Buffer OPAMP, implemented with a standard library cell in unity-gain configuration for voltage readout.

### 3.2. Channel Block Diagram

**Figure 2:**
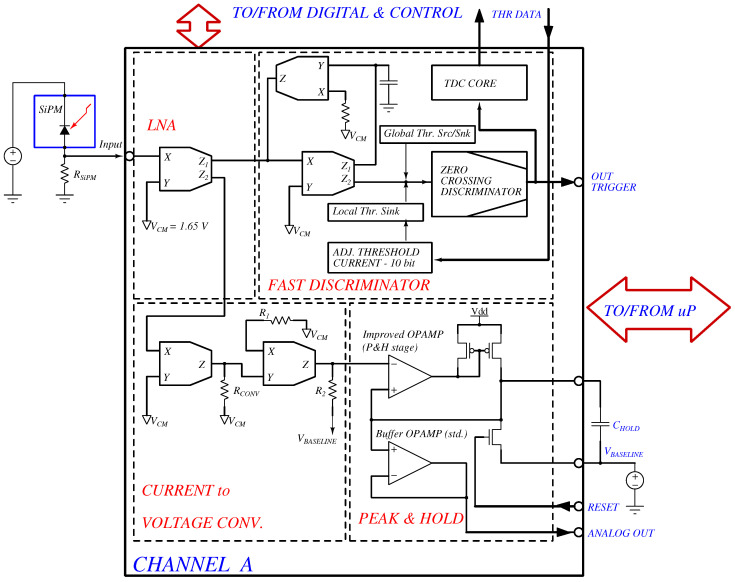
Functional block diagram of a single analog channel with LNA (CCII^+^-based) input, fast discriminator (pile-up reduction and zero-crossing discrimination with global/local threshold current injections), current-to-voltage converter with programmable resistors, and OPAMP-based peak-and-hold stage. VCM = internal mid-supply reference (≈1.65 V).

The functional structure of a single analog channel is shown in Figure 2.

It is divided into four main stages:

- **Low-Noise Input (LNA)**—Implemented with a CCII^+^ stage directly connected to the SiPM at its X terminal. In this configuration, the CCII^+^ acts as a current buffer, providing unit current gain and very low input impedance, while offering two parallel current outputs. This ensures faithful current-mode reception from the sensor and simultaneous driving of the fast discriminator and the peak-and-hold chain.
- **Fast Discriminator**—Realized with two cascaded CCII^+^ blocks configured as an integrator differentiator pair. In this topology, the first CCII^+^ reproduces the SiPM current pulse with low input impedance, while the second CCII^+^ operates in active feedback, effectively subtracting the slow recovery component and enhancing the sharp rising edges associated with individual photon events. This provides efficient pile-up reduction when signals occur at intervals comparable to the SiPM microcell recharge time.
  At the discriminator input node, programmable current injections are applied: global source/sink threshold DACs for coarse bias control, and a local sink DAC that defines the fine per-channel current threshold. This arrangement ensures reliable discrimination even at very low thresholds (down to the single-photon equivalent). The resulting current is then processed by a zero-crossing current comparator that generates the channel trigger.
- **Current-to-Voltage Converter (C/V)**—The secondary output of the input CCII^+^ is routed to a dedicated CCII^+^ stage operating as a current-to-voltage converter. Its feedback network includes three configurable resistors (Rconv, R1, R2), selectable through on-chip transmission gates, which allow tuning of conversion gain and dynamic range.

Current-to-Voltage conversion (left CCII^+^): $V_{\mathrm{iv}}=I_{\mathrm{in}}\cdot R_{\mathrm{CONV}}$

Voltage gain stage (right CCII^+^): $V_{\mathrm{out}}=V_{\mathrm{iv}}\cdot \left(R_{2}/R_{1}\right)$

Overall transimpedance with baseline offset:

(1) $$V_{\mathrm{out}}=V_{\mathrm{BASELINE}}+I_{\mathrm{in}}\cdot R_{\mathrm{CONV}}\cdot \left(R_{2}/R_{1}\right)$$

A summary of selectable values is reported in Table 1.

- **Peak-and-Hold (P&H)** The voltage output is captured by a peak-and-hold circuit based on the Improved OPAMP, optimized for fast charging of the hold capacitor (500 pF, external). The reset operation is performed by discharging the capacitor through a dedicated μP-controlled NMOS switch, while a unity-gain Buffer OPAMP isolates the hold node and ensures accurate interfacing to the external ADC under capacitive loading.
  The adopted peak-and-hold topology follows the classical architecture widely used in mixed-signal front-ends, as formalized in the analysis presented in [12]. This solution provides predictable settling, controlled charge injection, and reliable peak reconstruction for energy measurement.

### 3.3. SiPM Modeling

A behavioral model of the Hamamatsu S10362-11-050 (400-pixel) SiPM was implemented in Cadence to generate realistic transient inputs for circuit-level simulations. While similar pixel-branch models and comprehensive physical descriptions of SiPM operation have been presented in the literature [13,14], the present implementation follows our specific modeling framework described in [15].

**Figure 3:**
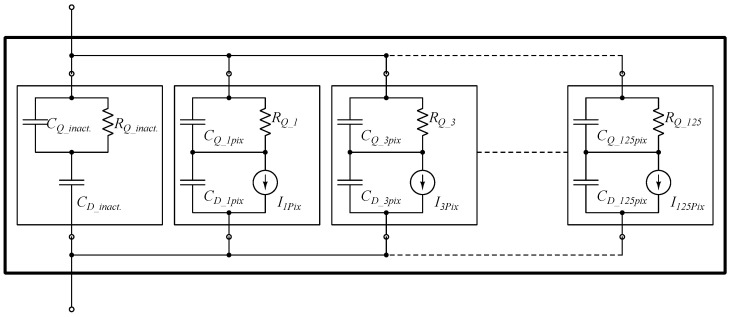
Behavioral SiPM model (Cadence).

The model uses a parallel-branch structure: each branch emulates the avalanche of *n* pixels (*n* = 1, 3, 10, 20, 40, 50, 75, 125). An inactive branch represents the remaining pixels as a lumped capacitive load, ensuring proper charge redistribution and recovery dynamics; see Figure 3.

Because the network is linear, higher counts (e.g., 200 pixels) are obtained by simultaneous activation of multiple branches (e.g., 125 + 75), without a dedicated “200-pixel” branch.

Each active branch includes a quenching resistor $R_{Q}$, a parasitic capacitance $C_{Q}$, a junction/depletion capacitance $C_{D}$, and an avalanche current source $I_{\mathrm{pix}}\left(t\right)$ gated by a 0–1 V control signal (see Table 2). The avalanche waveform $I_{\mathrm{pix}}\left(t\right)$ is modeled as a trapezoid with a 10 ps rise time, a 31 ps flat-top (10–41 ps), and a 10 ps fall time back to zero (41–51 ps).

With a peak amplitude of 2.93 mA per pixel (the total peak current scales with the number of coincident pixels *n*), the resulting charge is $Q\approx 1.2\times 10^{-13}\;C$ (≈120fC) per pixel, corresponding to a gain of approximately $7.5\times 10^{5}$ electrons, consistent with Hamamatsu datasheet values.

Transient simulation was performed by generating nine pulses starting at 10 ns with 100 ns spacing, enabling one branch at a time (1, 3, 10, 20, 40, 50, 75, 125 px), and finally a combined event of 125 px + 75 px (200 px) occurring 100 ns after the 125-px pulse. The resulting current was converted to voltage across a 50 Ω load.

Peak amplitudes at the typical corner range from ≈1 mV to ≈200 mV and show less than 2% deviation from ideal proportional scaling.

**Figure 4:**
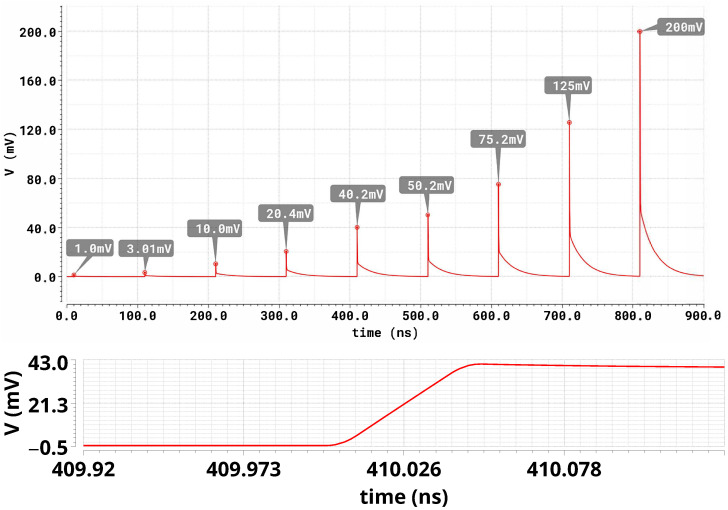
Simulated voltage waveforms on 50 Ω for different numbers of fired pixels. (**Top**): full waveform. (**Bottom**): zoomed view around the peak region of the fifth pulse in the sequence.

The simulated voltage waveforms obtained under these excitation conditions are shown in Figure 4, which reports the responses on 50 Ω for 1, 3, 10, 20, 40, 50, 75, 125, and 200 pixels (100 ns spacing). The sharp features observed in the simulated waveforms are a direct consequence of the fast SiPM-like current excitation adopted in the simulations, characterized by a rise time of approximately 50 ps.

The peak values annotated in the figure confirm the expected proportional scaling with the number of active pixels.

## 4. Key Building Blocks

### 4.1. Low-Noise CCII^+^: Core Building Block

The input stage of each analog channel is based on a custom CMOS CCII^+^, developed from standard current-mirror structures and subsequently optimized in [16]. Further refinements for SiPM readout were introduced by the authors in [17], where the architecture was adapted and validated in AMS 0.35 µm technology.

**Figure 5:**
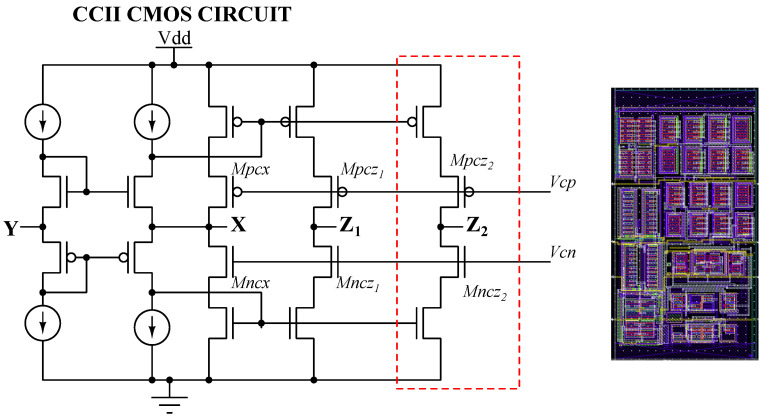
Simplified MOS-level schematic of the custom CCII^+^, together with its layout view shown on the right. For clarity, current sources replace slave transistors in the schematic. The dashed red box highlights the additional Z-output branch present in the dual-output (Z1/Z2) CCII^+^ variant. The physical layout footprint is 86μm×164μm in the AMS 0.35 µm technology, illustrating the achieved block area.

Two CCII^+^ variants are used: a single-output (Z) and a dual-output (Z1/Z2) version to feed the discriminator path and the P&H chain in parallel. Beyond cascoding the bias mirrors, additional fixed-bias cascode transistors, whose gates are driven by bandgap-derived reference voltages, are inserted on both the X and Z branches; they raise branch $r_{0}$, confine $V_{\mathrm{DS}}$ swings, and improve current replication. As a result, the X terminal remains stiff (very low effective input resistance) while Z1/Z2 present a high-$r_{0}$ current output. The block operates from 3.3 V with a mid-supply reference $V_{\mathrm{CM}}=1.65\;V$ for internal biasing. The schematic and its corresponding layout view are shown in Figure 5. The layout footprint is 86μm×164μm, highlighting the compactness of the block in AMS 0.35 µm CMOS.

Simulation results confirm the effectiveness of the CCII^+^ implementation under realistic conditions, evaluated by small-signal current excitation at node X and observation of the output response.

**Figure 6:**
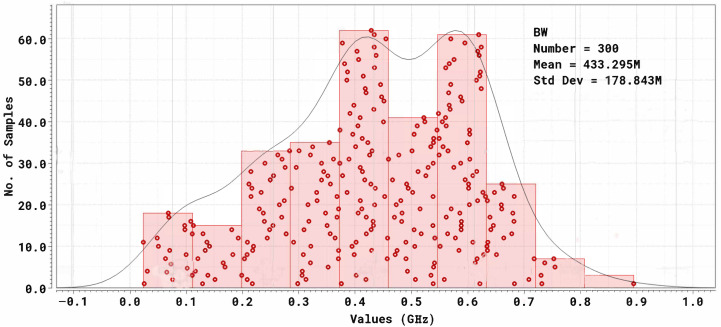
AC magnitude response with Monte Carlo simulation (300 samples, process + mismatch). Mean bandwidth: 433 MHz; standard deviation: σ=179MHz.

The −3dB bandwidth was first estimated from AC analyses and then validated through a Monte Carlo simulation with 300 samples (process + mismatch). The resulting distribution, shown in Figure 6, yields a mean value of 433.3 MHz with a standard deviation of 178.8 MHz, consistent with the design target for wideband current-mode SiPM readout.

The number of Monte Carlo samples was selected as a practical trade-off between statistical representativeness and simulation time. The estimated mean value and standard deviation of the −3dB bandwidth were observed to remain stable when increasing the number of iterations, indicating that 300 samples are sufficient for the intended statistical characterization.

The observed dispersion is mainly driven by the sensitivity of the CCII^+^ bandwidth to transconductance and parasitic capacitance variations in this technology.

The input impedance magnitude $|Z_{\mathrm{in}}\left(f\right)|$ at node X is about 19.8Ω at low frequency (≈1kHz), rising with frequency and reaching ∼300Ω around 288–300 MHz, in line with the intended low-impedance coupling to the SiPM. The input impedance was extracted from AC simulations by applying a small AC voltage excitation at node X and evaluating the ratio between voltage and input current as a function of frequency.

**Figure 7:**
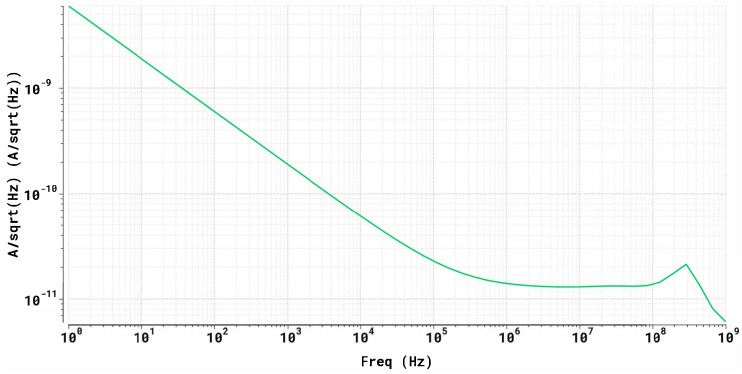
Output-referred current-noise spectral density at the CCII^+^ output node (1 Hz–1 GHz).

Noise was evaluated with Spectre AC-noise at the LNA output node by integrating the simulated output-referred noise spectral density over the 1 Hz–1 GHz band, yielding the RMS current noise $I_{n,\mathrm{rms}}=\sqrt{\int_{1\;\mathrm{Hz}}^{1\;\mathrm{GHz}}S_{i}\left(f\right)\;df}$. The resulting output-referred current noise is 414 nA RMS. Compared to the minimum single-photon equivalent transient signal of 2.5μA peak, this corresponds to an SNR of 6.0. These results are illustrated in Figure 7, which shows the output-referred current-noise spectral density.

### 4.2. Fast Zero-Crossing Current Comparator

The fast discriminator in each channel employs a CMOS current comparator derived from the topology introduced by Ziabakhsh et al. [18], chosen for its low input impedance, nanosecond-scale response, and reduced static power consumption.

In the proposed architecture, this block implements the zero-crossing discriminator function shown in the system-level diagram of Figure 2.

**Figure 8:**
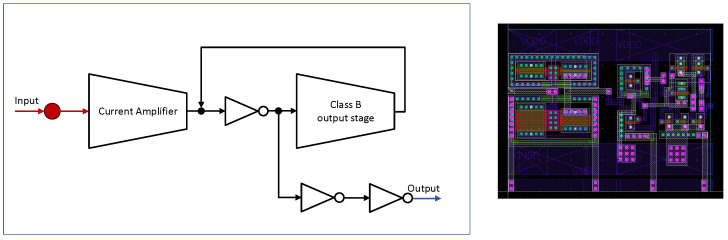
Functional block diagram of the fast zero crossing CMOS current comparator used in the zero crossing discriminator (**left**) together with its layout implementation in the AMS 0.35 µm process (**right**). The layout footprint is 33μm×27μm, illustrating the compactness of the block.

Without reproducing the full transistor-level schematic reported in the original work, Figure 8 presents the functional structure implemented in this ASIC, which preserves the same architectural partitioning: (i) a current amplifier stage based on dual current mirrors to provide gain for low-level SiPM signals; (ii) a stage composed of an intermediate inverter and a class-B output stage, enabling fast switching with low static power consumption; and (iii) a final chain of CMOS inverters that sharpens the transition and drives the subsequent digital logic.

The intermediate inverter and the class-B stage operate with positive feedback, so that small input-current variations are rapidly converted into a sharp voltage transition before digital buffering.

The input node of the comparator collects the signal current delivered by the CCII^+^ stage together with programmable static bias currents (global and local), which define the effective discrimination threshold. The comparator triggers when the signal current exceeds the summed bias contributions.

For the present implementation, transistor dimensions were resized to match the current levels delivered by the preceding CCII^+^ stages and to achieve the desired speed–noise tradeoff in the AMS 0.35 µm process. Only functional modifications were introduced, without altering the structural organization of the original comparator. Simulated propagation delays fall in the sub-nanosecond to low-nanosecond range across the full 1–200 px dynamic range of the SiPM model, fully adequate for fast zero-crossing detection in high-rate environments.

### 4.3. Discriminator Pulse-Width Shaping Circuit

**Figure 9:**
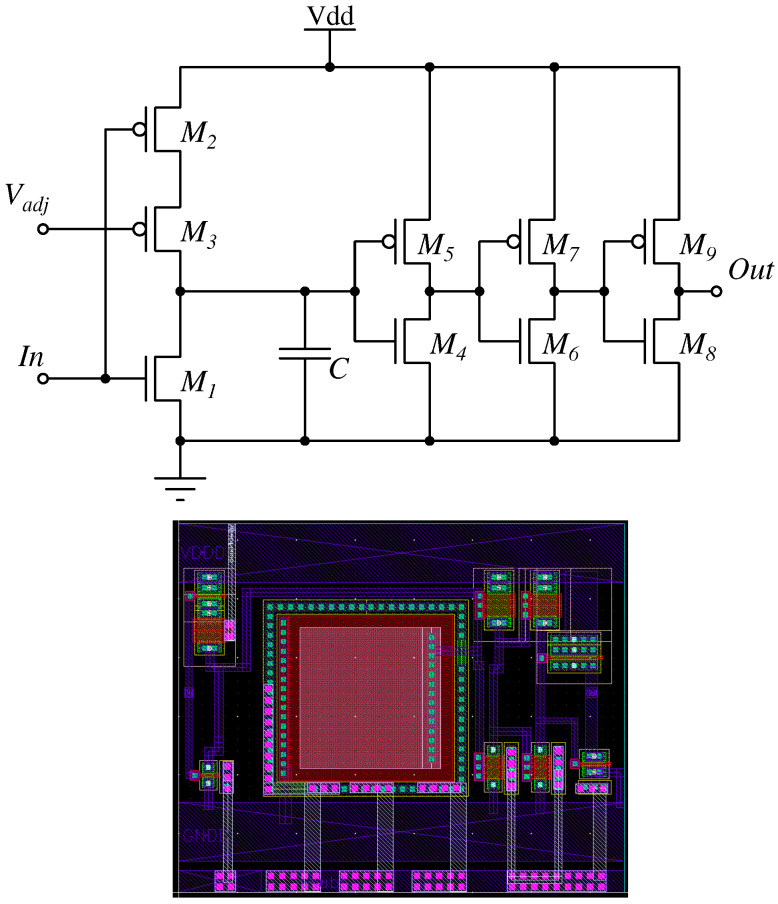
Transistor-level schematic (**top**) and physical layout (**bottom**) of the programmable pulse-width shaping circuit implementing a MOS current steering monostable.

To suppress spurious re-triggering and ensure a clean digital output under high-rate conditions, each discriminator is followed by a compact MOS monostable that enforces a programmable minimum pulse width. The input signal “In” corresponds to the digital output of the preceding fast current comparator (zero-crossing discriminator) and is processed by the pulse-width shaping circuit to enforce a programmable minimum pulse width and suppress spurious re-triggering. The detailed transistor-level schematic and the corresponding layout are shown in Figure 9. The circuit operates by rapidly discharging a small storage capacitor *C* at the rising edge of the discriminator signal, followed by a controlled recharge driven by a programmable current supplied by the global shaping DAC.

Transistors M1–M2 form the fast discharge/recharge pair. During the idle phase, M2 (PMOS) keeps the capacitor charged, while M1 (NMOS) remains off. When a discriminator pulse arrives, M1 turns on and forces a rapid discharge of *C*, initiating the monostable cycle. The recharge slope is set by M3 (PMOS), biased through a dedicated DAC output ($V_{\mathrm{adj}}$), and operating in saturation to provide a stable, tunable charging current. The bias voltage $V_{\mathrm{adj}}$ is generated by one of the on-chip current DACs described in Section 4.4 and distributed through dedicated master/slave current-mirror chains. As the voltage across *C* rises, the three cascaded CMOS inverter pairs (M4–M9) decode the analog ramp into a regenerated digital transition, returning the shaped output to logic low once the programmed threshold is reached. The corresponding transistor dimensions are reported in Table 3.

This mechanism produces a clean, monotonic digital pulse with a width that is fully determined by the programmable recharge current. Across AMS 0.35 µm typical-corner simulations, the discriminator rising edge introduces a fixed delay of approximately 2 ns regardless of DAC settings. The programmable pulse extension spans a wide range: with all DAC bits enabled, the pulse width extends by approximately 6.5–7 ns beyond the input edge; enabling only the MSB yields a comparable extension of 6.6 ns, while enabling only the LSB produces the longest extension, approximately 866 ns. Intermediate codes provide continuous control of the pulse width over this entire range. This programmability also provides effective suppression of double-triggering phenomena originating from SiPM afterpulsing and partial microcell recovery, ensuring clean discriminator operation even under high-rate conditions.

### 4.4. Master Bias and Current DACs

A dedicated subsystem provides stable biasing and programmable current references to the entire ASIC. A Master Bias Generator produces a temperature- and process-compensated reference current, which is routed to global and per-channel nodes through a set of binary-weighted current–splitting DACs. Configuration words are written through an SPI interface into a 10-bit Register Bank, which controls all programmable bias points in the device. The Master Bias block includes an internal kickstart mechanism to ensure correct startup, preventing convergence to the undesired zero-current equilibrium that is intrinsic to self-biased current generators.

Two global 10-bit DACs generate the positive and negative discriminator threshold currents, enabling offset cancellation and precise control of the trigger level. Additional global DACs set the bias currents of the CCII^+^ stages (nominally 150 µA) and define the current used for pulse-width shaping in the discriminator, allowing controlled pulse extension for improved stability. Each analog channel includes its own 10-bit local threshold DAC, enabling fully independent calibration down to the single-photon equivalent level.

**Figure 10:**
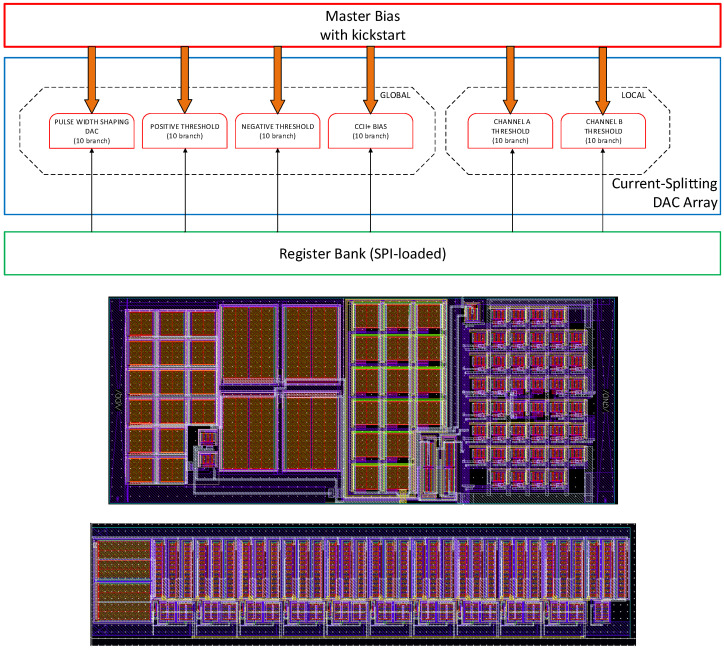
Functional block diagram of the biasing and threshold-generation subsystem (**top**), layout of the Master Bias Generator (**middle**), and layout of one 10-branch current-splitting DAC (**bottom**). Configuration words are loaded through an SPI-driven 10-bit Register Bank, which controls the binary-weighted splitters. The Master Bias includes a kickstart mechanism to avoid the undesired zero-current stable point typical of self-biased generators. Global DACs provide positive and negative discriminator thresholds, CCII^+^ bias currents, and a programmable pulse-width shaping current, while local DACs independently define the per-channel threshold settings.

The design follows the current-splitting approach introduced by Delbrück and Van Schaik [19], where binary-weighted branching provides compact, accurate, and layout-efficient current generation. In this implementation, all DACs adopt a 10-branch splitter structure, achieving a resolution of approximately 200 nA per LSB with minimal area overhead. Figure 10 illustrates the functional structure of the subsystem, together with the physical layouts of the Master Bias Generator and of one representative splitter DAC.

## 5. Digital Control, Configuration and Readout

Each acquisition channel incorporates an 11-bit time marker generated by a segmented delay-line Time-to-Digital Converter (TDC). The discriminator edge propagates through a chain of progressively delayed taps, and the index of the active tap encodes the arrival time with fine granularity. The discriminator output triggers the delay line, while an external microcontroller (µP) provides the system clock for coarse synchronization. On chip, the discriminator edge is stretched to approximately 13 ns to guarantee reliable sampling of all delay segments under normal operating conditions. The resulting 11-bit word is latched and made available to the readout logic.

In this first prototype the segmented-delay TDC is included mainly to validate the integration of a compact timing block alongside the current-mode analog front end. The present design does not yet implement full real-time TDC management (e.g., multi-event buffering or advanced timing reconstruction), but demonstrates correct triggering, stable propagation along the segmented line, and coexistence with the fully integrated analog chain. A complete timing engine is foreseen for the next-generation ASIC, which is planned in a more advanced 0.18 µm CMOS node.

The two channels share a Digital Control and Readout block that also acts as the interface to the µP via a standard SPI link. 10-bit configuration registers provide full programmability of the analog chain, including:

- fine control of the discriminator thresholds through local 10-bit current DACs;
- global source/sink bias currents distributed to all channels;
- dedicated bias currents for the CCII^+^ stages (nominally 150 µA per unit);
- adjustable injection currents for signal shaping and pulse-width control.

This level of programmability enables precise control of detection sensitivity and operating points, while maintaining robustness against mismatch and offsets. It also ensures complete compatibility with external digital platforms, despite the reduced pin count required by the miniaturized ASIC.

TDC data (time tags) are serialized and buffered before transmission to the µP.

Overall, the digital subsystem complements the analog front end—designed to replace all discrete analog circuitry used in previous detector generations—by providing configuration, bias distribution, threshold control and basic timing capture in a compact mixed-signal architecture. This first implementation establishes the functional feasibility of the approach and sets the foundation for a future, fully integrated timing solution in more advanced technologies.

## 6. Results

### 6.1. Simulation: Fast Discriminator and Pile-Up Reduction

**Figure 11:**
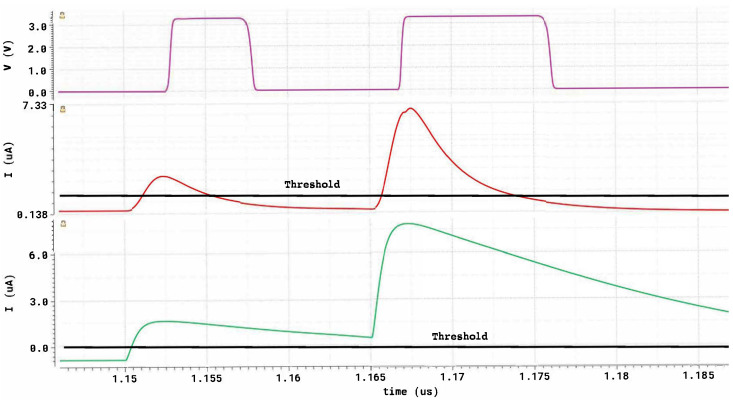
Simulated transient response of the fast discriminator chain under two closely spaced SiPM-like events (1 p.e. and 3 p.e., 15 ns separation). **Bottom**: LNA + CCII^+^ output signal current. **Middle**: output current after the pile-up reduction stage. **Top**: digital output of the high-speed current comparator. The horizontal line indicates the applied discrimination threshold.

Figure 11 illustrates the simulated transient behavior of the fast-discriminator chain under realistic SiPM excitation with two closely spaced events (1 p.e. and 3 p.e., separated by 15 ns).

The bottom trace shows the current delivered by the LNA + CCII^+^ input stage. Due to the microcell recovery tail of the first pulse, the second event remains partially masked by pile-up: only the first peak crosses the discrimination threshold (horizontal reference line), while the second does not.

The middle trace reports the signal after the dedicated pile-up reduction stage, implemented by a CCII^+^ with an active-feedback branch acting as a current differentiator. This processing suppresses the slowly varying recovery component while preserving the fast rising edge associated with each photon event. As a result, both the 1 p.e. and 3 p.e. pulses clearly cross the threshold despite their limited temporal separation.

The top trace shows the corresponding digital output of the high-speed current comparator. Thanks to the programmable threshold provided by the local 10-bit current DAC, clean timing edges are generated even for the minimum signal amplitude (1 p.e.), and the second event is correctly identified without distortion from the remnant tail of the first pulse.

These results confirm that the discriminator remains fully operational at high rate and under significant pile-up conditions.

### 6.2. Simulation: Peak-and-Hold (P&H) and Discriminator Threshold Effects

The Peak-and-Hold (P&H) stage was functionally verified through detailed transient simulations consisting of two burst sequences with increasing SiPM amplitudes. Two acquisition bursts were generated, each consisting of eight pulses with increasing amplitude, corresponding to coincident pixel counts ranging from 1 to 200. In this setup, the SiPM current was converted to voltage across an external 100 Ω load resistor and AC-coupled into the chip through a 2.2 nF capacitor. This configuration reproduces the expected interface of practical detector hardware while allowing direct visualization of the raw SiPM-like waveform.

In the first burst, the discriminator threshold was programmed to its minimum value using the local 10-bit current DAC. All events produced valid triggers, from the smallest (1 p.e.)—visible as a ≈0.6 mV excursion in the zoomed view—to the largest pulses in the sequence (approaching ∼130 mV). The held voltage $V_{P\&H}$ faithfully followed the peak value of each pulse and remained stable throughout each acquisition window.

At 9 μs, a digital reset command discharged the hold capacitor. The reset signal is externally applied in the simulation testbench and its timing is chosen to illustrate the functional discharge of the hold capacitor and the effect of a dynamic threshold change within the same simulation run. Immediately after the reset, the threshold was raised to its maximum value: as expected, no discriminator pulses were generated in the second burst, including those associated with the highest-amplitude events. This confirms full control of the detection sensitivity through the programmable threshold mechanism.

**Figure 12:**
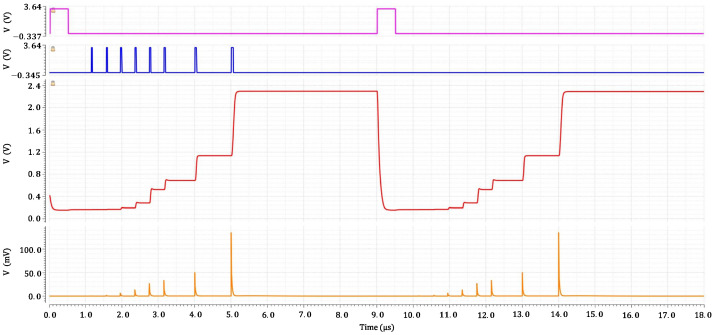
Transient functional verification of the Peak-and-Hold (P&H) stage obtained within a single simulation run. Two sequences of SiPM-like pulses with increasing amplitude (1–200 coincident pixels) are applied to the input. (bottom) SiPM-like voltage obtained across a 100 Ω load resistor and AC-coupled into the chip; (next) held voltage *V*_P&H_ at the output pad, showing correct peak tracking and hold behavior; (next) discriminator trigger pulses; (top) externally applied digital reset signal used in the simulation testbench to discharge the hold capacitor. In the first sequence, the discriminator threshold is set to its minimum value and all events generate valid triggers. After the externally applied reset at 9 μs, the threshold is dynamically raised to its maximum value, and no discriminator pulses are generated in the subsequent sequence.

**Figure 13:**
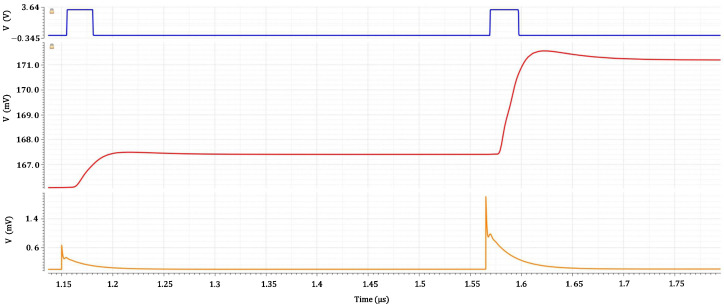
Zoomed view of the first two pulses of the first burst (1 p.e. and 3 p.e.). The SiPM-like voltage shows a ≈0.6 mV peak for the single-photon event and a slightly larger excursion for the 3 p.e. pulse. The held voltage $V_{P\&H}$ correctly captures the peak of each event, and the discriminator produces clean threshold crossings at the programmed low threshold.

Figure 12 and Figure 13 summarize the results. The overall waveform shows proper peak tracking, clean discriminator behavior, and stable hold operation, while the zoomed view highlights the first two pulses (1 p.e. and 3 p.e.), clearly resolved in amplitude despite their small magnitude at the input.

### 6.3. Chip Layout and Physical Implementation

The ASIC was implemented in AMS 0.35 µm CMOS using a compact pad-limited floorplan. The final die measures 2.31 × 2.31 mm^2^ (∼5.34 mm^2^) and integrates all analog, mixed-signal and digital subsystems required for complete two-channel SiPM readout.

The physical layout is organized into clearly separated functional regions. The two analog front-end channels are placed symmetrically to minimize mismatch and ensure identical signal paths. The digital control and SPI interface are located near the padframe to reduce coupling toward the sensitive analog blocks.

Particular attention was devoted to substrate and ground-noise mitigation. Analog and digital grounds are routed separately and tied together only at a single star-point near the padframe. Two distinct guard-ring structures (analog and digital semirings) fully surround their respective regions, reducing substrate coupling and preventing injection of switching noise from the digital block into the high-gain analog front end. Critical analog nets—including the CCII^+^ stages and the discriminator input—are routed with dedicated metal layers and local shielding.

**Figure 14:**
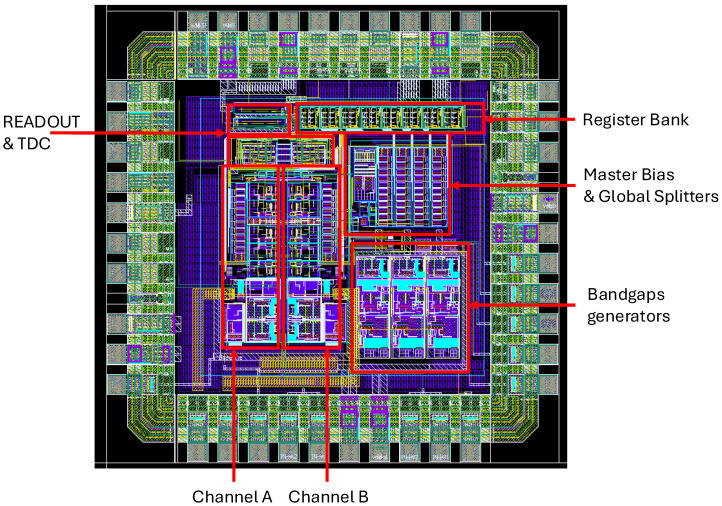
Final layout of the dual-channel mixed-signal ASIC in AMS 0.35 µm CMOS. Highlighted regions correspond to: (i) the two analog front-end channels, (ii) the centralized master-bias generator and current-splitting DACs, (iii) the digital subsystem; three dedicated on-chip bandgap reference blocks are also included to generate the mid-supply and cascode bias voltages required by the CCII^+^ stages. Separate analog and digital ground networks, together with dedicated analog/digital semiring guard structures, minimize substrate noise injection and ensure robust mixed-signal operation. The die area is 2.31 × 2.31 mm^2^ (∼5.34 mm^2^) for a 5 × 5 mm^2^ QFN32 package.

Figure 14 shows the complete layout of the ASIC. Highlighted regions indicate the two analog channels, the centralized bias and DAC network, and the digital subsystem; the layout also includes three dedicated on-chip bandgap reference blocks that generate the mid-supply reference ($V_{\mathrm{CM}}\approx$ 1.65 V) and two cascode bias voltages for the PMOS and NMOS branches, respectively, required by the CCII^+^ stages of the two analog channels. The organization confirms the feasibility of integrating a current-mode analog front end together with basic timing and configuration logic within a compact mixed-signal ASIC.

## 7. Conclusions

A dual-channel mixed-signal ASIC for SiPM readout has been presented and validated through extensive circuit-level simulations in AMS 0.35 µm CMOS technology. The design integrates low-impedance CCII^+^ stages for current-domain processing, a fast discriminator chain with effective pile-up reduction, and a Peak-and-Hold architecture for accurate pulse-amplitude capture over a wide dynamic range. Programmable biasing and threshold control are implemented through compact current-splitting DACs, enabling fine adjustment of operating points and single-photon sensitivity. Simulation results confirm full functional correctness of all analog and digital blocks, with an analog bandwidth exceeding 400 MHz, an input-referred SNR of approximately 6 for single-photon events, robust pile-up rejection, and a per-channel power consumption of 19.5 mW at 3.3 V. The ASIC has been submitted for fabrication in a compact QFN32 5 × 5 mm^2^ package, with a die area of 2.31 × 2.31 mm^2^, providing a fully integrated solution intended to replace discrete analog front-end electronics used in previous detector generations. This prototype establishes the viability of a fully programmable current-mode SiPM front end and represents the foundation for future developments toward higher integration and enhanced timing performance in space-borne and compact detector applications.

## Figures and Tables

**Table 1:** Programmable resistor values for the current-to-voltage converter ($R_{\mathrm{CONV}}$, $R_{1}$, $R_{2}$) used to adjust channel gain and dynamic range.

Element	Bits	Selectable Values (Ω)
$R_{\mathrm{CONV}}$	2	500, 1 k, 1.5 k, 2 k
$R_{1}$	2	500, 1 k, 1.5 k, 2 k
$R_{2}$	3	5 k, 25 k, 45 k, 50 k, 65 k, 80 k, 105 k

**Table 2:** Per-pixel parameters used in the SiPM behavioral model.

Parameter	Symbol	Value (per Pixel)
Junction capacitance	$C_{D}$	90 fF
Quenching resistor	$R_{Q}$	200 kΩ
Parasitic capacitance	$C_{Q}$	5 fF

**Table 3:** Transistor dimensions of the pulse-width shaping circuit (AMS 0.35 µm).

Device	Type	W/L (µm/µm)
M1	NMOS	1/0.35
M2	PMOS	2/0.35
M3	PMOS	2/2
M4	NMOS	1/2
M5	PMOS	2/2
M6	NMOS	1/2
M7	PMOS	2/2
M8	NMOS	2/0.35
M9	PMOS	4/0.35

## Data Availability

The data presented in this study are available on request from the corresponding author.
